# A Bayesian Multivariate Gametic Model in a Reciprocal Cross with Genomic Information: An Example with Two Iberian Varieties

**DOI:** 10.3390/ani13101648

**Published:** 2023-05-16

**Authors:** Houssemeddine Srihi, David López-Carbonell, Noelia Ibáñez-Escriche, Joaquim Casellas, Pilar Hernández, Sara Negro, Luis Varona

**Affiliations:** 1 Facultad de Veterinaria, Instituto Agrolimentario de Aragón (IA2), Universidad de Zaragoza, 50013 Zaragoza, Spain; houssemsrihi@unizar.es (H.S.);; 2 Institute for Animal Science and Technology, Universitat Politècnica de València, 46022 Valencia, Spain; 3 Department of Animal and Food Science, Universitat Autònoma de Barcelona, 08193 Barcelona, Spain; 4 Programa de Mejora Genética “Castúa”, INGA FOOD S.A. (Nutreco), 06200 Almendralejo, Spain

**Keywords:** gametic model, Iberian pig, crossbreeding, Retinto, Entrepelado

## Abstract

**Simple Summary:**

INGA FOOD, S.A. initiated a crossbreeding program involving two Iberian pig varieties: Retinto and Entrepelado. The primary objective of this program is to produce an F1 hybrid sow that exhibits enhanced reproductive performance. In a previous investigation, variations in the reproductive performance of sows, specifically litter size, were observed among the reciprocal crosses. These variations indicate the presence of genomic imprinting effects. To assess the influence of genetic origin, we developed a multivariate gametic model to estimate the gametic correlations between paternal and maternal effects. Gametic correlations lower than one could potentially explain the performance differences observed across the reciprocal crosses. Despite having limited data, the study’s findings suggest that the gametic correlation estimate between paternal and maternal effects on litter size is lower in the Entrepelado population compared to the Retinto population.

**Abstract:**

INGA FOOD, S.A. initiated a crossbreeding program between two Iberian pig varieties, Retinto (R) and Entrepelado (E), with the goal of producing a hybrid sow (F1). Several studies have been conducted to evaluate its productive performance, and these studies have revealed differences in litter size between the two reciprocal crosses, suggesting the presence of genomic imprinting effects. To further investigate these effects, this study introduces a multivariate gametic model designed to estimate gametic correlations between paternal and maternal effects originating from both genetic backgrounds involved in the reciprocal crosses. The dataset consisted of 1258 records (the total number born—TNB and the number born alive—NBA) from 203 crossbred dams for the Entrepelado (sire) × Retinto (dam) cross and 700 records from 125 crossbred dams for the Retinto (sire) × Entrepelado (dam) cross. All animals were genotyped using the GeneSeek^®^ GPP Porcine 70 K HDchip (Illumina Inc., San Diego, CA, USA). The results indicated that the posterior distribution of the gametic correlation between paternal and maternal effects was distinctly different between the two populations. Specifically, in the Retinto population, the gametic correlation showed a positive skew with posterior probabilities of 0.78 for the TNB and 0.80 for the NBA. On the other hand, the Entrepelado population showed a posterior probability of a positive gametic correlation between paternal and maternal effects of approximately 0.50. The differences in the shape of the posterior distribution of the gametic correlations between paternal and maternal effects observed in the two varieties may account for the distinct performance outcomes observed in the reciprocal crosses.

## 1. Introduction

The Iberian breed is widely renowned for its ability to produce some of the highest-quality pork [1]. This breed is particularly well-adapted to the “Dehesa” environment in southwestern Spain, which is characterized by a savannah landscape and is composed of grass, cork, and holm oaks with seasonal production. Traditionally, Iberian pig production was dominated by purebred varieties and extensive management practices. However, in recent decades, there has been a shift toward more intensive farming practices that incorporate crossbreeding with Duroc boars to improve growth and efficiency at commercial stages [2].

The regulatory norms for Iberian pig production allow crossbreeding, as long as the sow is of purebred Iberian stock. The reproductive performance of the Iberian sows is lower than that of white pig populations [3], which is a major limitation of its use in intensive farms. Therefore, improvement in the reproductive efficiency of Iberian sows is crucial for their economic sustainability. Several studies have identified genetic variability for prolificacy within and between varieties of Iberian pig [4,5]. To take advantage of this variability, the INGA FOOD, S.A. company has developed a crossbreeding scheme between two Iberian varieties (Retinto and Entrepelado) to generate an F1 hybrid sow, which exhibits significant heterosis for litter size [6]. However, this study also found differences in the reproductive performance between the two reciprocal crosses (Entrepelado × Retinto, ER, vs. Retinto × Entrepelado, RE), suggesting that these differences may be attributed to parental imprinting [7] (i.e., the effects from alleles may differ whether they are transmitted by paternal or maternal gametes). In fact, there is increasing evidence of the importance of imprinting in placenta development [8], and certain imprinted genes have been proposed as candidates for pig litter size [9].

In recent years, some algorithms have been proposed to develop a genomic analysis of imprinting [10] from the genomic information provided by commercial genotyping devices. However, knowledge of the parental haplotype phase of the SNP markers is required to differentiate the paternal or maternal gametic effects. Some approaches have been developed to reconstruct haplotype phases [11].

Phenotypic information from reciprocal crosses offers the opportunity to compare the paternal and maternal effects of each parental population. In the absence of imprinting, the correlation between the paternal and maternal effects from the same population should be one. Imprinting, on the other hand, results in a lower correlation. Accordingly, the goal of this study was to apply the multivariate gametic model developed in a previous study [12] that utilizes genomic information and is capable of estimating the paternal and maternal gametic contributions of Retinto and Entrepelado varieties in the ER and RE crosses, along with their correlations.

## 2. Materials and Methods

**Phenotypic and Genomic Data.** The phenotypic data used in this study consisted of the total number born, TNB, and the number of piglets born alive, NBA, in 203 ER and 125 RE sows. The ER sows were the offspring of 38 purebred Entrepelado boars and 139 Retinto dams, whereas the RE sows were generated from 38 Retinto boars and 92 Entrepelado dams. A summary of the data is presented in Table 1.

Genotyping was performed with the GeneSeek^®^ GPP Porcine 70 K HDchip (Illumina Inc., San Diego, CA, USA) on all ER and RE crossbred sows, as well as on 341 Retinto and 350 Entrepelado purebred individuals. Due to shared purebred ancestors, there was some degree of relationship between a subset of the ER and RE crossbred sows and the purebred individuals, although not all of them were genotyped. The original genotype data consisted of 60,224 autosomal SNPs, which were filtered by excluding SNP markers with a call rate below 0.90 and a minor allele frequency lower than 0.05 in each population. Among these, 4212 were discarded due to a call rate lower than 0.90, 11,234 were found to be monomorphic, and 9876 and 11,516 had a minor allele frequency lower than 0.05 in the Entrepelado and Retinto populations, respectively. Finally, a total of 23,386 SNPs were retained.

**Haplotype Phasing.** AlphaPhase software [11] was used for each chromosome separately, utilizing genotypes of both crossbred and purebred individuals, as well as a pedigree of 1601 individuals. AlphaPhase was executed with a tolerance of 1% of genotype errors and 1% disagreement between genotypes and haplotypes. The number of surrogates and percentage of surrogate disagreement was set to 10. Nine different scenarios were applied with core lengths of 75, 100, and 125 SNPs and tail lengths of 100, 150, and 200 SNPs (see Table 2). The scenarios were evaluated for concordance, and haplotype assignments that coincided in seven or more scenarios were retained for subsequent analysis.

**Statistical Model.** Once the haplotype phases were calculated, data were analyzed with the model proposed by Shiri et al. [12]:

$$y_{\left(ER\right)}=X_{\left(ER\right)}b_{\left(ER\right)}+B_{\left(ER\right)}s_{\left(ER\right)}+Z_{\left(ER\right)}p_{\left(E\right)}+W_{\left(ER\right)}m_{\left(R\right)}+e_{\left(ER\right)}\;y_{\left(RE\right)}=X_{\left(RE\right)}b_{\left(RE\right)}+B_{\left(RE\right)}s_{\left(RE\right)}+Z_{\left(RE\right)}p_{\left(R\right)}+W_{\left(RE\right)}m_{\left(E\right)}+e_{\left(RE\right)}$$

In this equation, $y_{\left(ER\right)}$ and $y_{\left(RE\right)}$ refer to the vectors of phenotypic records (TNB or NBA) for the ER and RE crosses, respectively. The terms$\;b_{\left(ER\right)}\;\mathrm{and}\;b_{\left(RE\right)}$ correspond to systematic effects, and $s_{\left(ER\right)}$ and $s_{\left(RE\right)}$ represent the permanent sow environmental effects. Paternal effects for the Entrepelado (E) and Retinto (R) populations are denoted by $p_{\left(E\right)}$ and $p_{\left(R\right)}$, respectively. Maternal effects for the Entrepelado (E) and Retinto (R) are represented by $m_{\left(E\right)}$ and $m_{\left(R\right)}$. Additionally, $e_{\left(ER\right)}$ and $e_{\left(RE\right)}$ are the residual effects for the ER and RE crosses, respectively. The systematic effects vectors included the order of parity with five levels (first, second, third, fourth, and fifth or more) and herd–year–season with thirty-four levels. Further,$X_{ER},X_{RE},B_{ER},B_{RE},Z_{ER},Z_{RE},W_{ER},\;\mathrm{and}\;W_{RE}$ are the corresponding incidence matrices.

Following [12], the prior distribution of the permanent sow environmental effects was:

$$\left[\begin{array}{c} s_{\left(ER\right)}\\ s_{\left(RE\right)} \end{array}\right]\sim N\left(\begin{array}{c} 0\\ 0 \end{array},I⊗S\right)$$

where:

$$S=\left[\begin{array}{cc} \sigma_{s\left(ER\right)}^{2} & 0\\ 0 & \sigma_{s\left(RE\right)}^{2} \end{array}\right]$$

where $\;\sigma_{s\left(ER\right)}^{2}\;$ and $\sigma_{s\left(RE\right)}^{2}\;$ are the variances of the permanent sow environmental effects for ER and RE, respectively. The prior distributions of the gametic effects for the Entrepelado (E) and Retinto (R) populations are:

$$\left[\begin{array}{c} p_{\left(E\right)}\\ m_{\left(E\right)} \end{array}\right]\sim N\left(\begin{array}{c} 0\\ 0 \end{array},G_{E}⊗V_{E}\right)\;\left[\begin{array}{c} p_{\left(R\right)}\\ m_{\left(R\right)} \end{array}\right]\sim N\left(\begin{array}{c} 0\\ 0 \end{array},G_{R}⊗V_{R}\right)$$

where:

$$V_{E}=\left[\begin{array}{cc} \sigma_{p\left(E\right)}^{2} & \sigma_{pm\left(E\right)}\\ \sigma_{pm\left(E\right)} & \sigma_{m\left(E\right)}^{2} \end{array}\right]$$

and:

$$V_{R}=\left[\begin{array}{cc} \sigma_{p\left(R\right)}^{2} & \sigma_{pm\left(R\right)}\\ \sigma_{pm\left(R\right)} & \sigma_{m\left(R\right)}^{2} \end{array}\right]$$

where $\sigma_{p\left(E\right)}^{2}$, $\sigma_{m\left(E\right)}^{2}$, and $\sigma_{pm\left(E\right)}\;$refer to the variances of the paternal and maternal gametic effects and the covariance between them, respectively, for the Entrepelado population. Similarly, $\sigma_{p\left(R\right)}^{2}$, $\sigma_{m\left(R\right)}^{2}$, and $\sigma_{pm\left(R\right)}$ represent the variances of the paternal and maternal gametic effects and the covariance between them, respectively, for the Retinto population. Additionally, $G_{E}$ and $G_{R}$ are the gametic relationship matrices of the Entrepelado or Retinto gametes, respectively, regardless of whether they are transmitted as paternal or maternal gametes. These matrices describe the relationships among the gametes from Entrepelado and Retinto origins, and they are calculated using the algorithm proposed by Nishio and Satoh [10]:

$$G_{E}=\frac{M_{E}M_{E}{}^{\prime }}{\sum_{i}^{N_{SNP}}q_{\left(E\right)i}\left(1-q_{\left(E\right)i}\right)}\;G_{R}=\frac{M_{R}M_{R}{}^{\prime }}{\sum_{i}^{N_{SNP}}q_{\left(R\right)i}\left(1-q_{\left(R\right)i}\right)\;}$$

where $M_{E}$ and $M_{R}$ are the matrices of the number of genotyped individuals (***n***) × the number of SNP ($N_{SNP}$), whose elements $M_{E}\left(i,j\right)$ (or $M_{R}\left(i,j\right)$ take the value $q{\left(E\right)}_{j}$ (or $q{\left(R\right)}_{j}$) or $-\left(1-q{\left(E\right)}_{j}\right)$ (or $-\left(1-q{\left(R\right)}_{j}\right)$), depending on whether the *jth* allele of the gametes transmitted for the *ith* individual is A_1_ or A_2_ and of Entrepelado (or Retinto) origin. Additionally, $q{\left(E\right)}_{j}$ and $q{\left(R\right)}_{j}$ represent the allelic frequencies of the A_2_ allele in the Entrepelado (E) and Retinto (R) populations, respectively. The prior distributions for the (co) variance components and the systematic effects were assumed to be flat. The analysis was performed using Bayesian inference with the Gibbs sampler [13] and implemented with Gibbsf90 software [14]. The analysis was performed using 10 million iterations after discarding the first million.

At each iteration of the Gibbs sampler, the (co) variances components samples were utilized to compute the samples from the marginal posterior distribution of the correlations between the paternal and maternal gametic effects for Entrepelado ($r_{pm\left(E\right)}$) and Retinto ($r_{pm\left(R\right)}$):

$$r_{pm\left(E\right)}=\frac{\sigma_{pm\left(E\right)}}{\sqrt{\sigma_{p\left(E\right)}^{2}\sigma_{j\left(E\right)}^{2}}}\;\mathrm{and}\;r_{pm\left(R\right)}=\frac{\sigma_{pm\left(R\right)}}{\sqrt{\sigma_{p\left(R\right)}^{2}\sigma_{j\left(R\right)}^{2}}}$$

## 3. Results and Discussion

**Figure 1:**
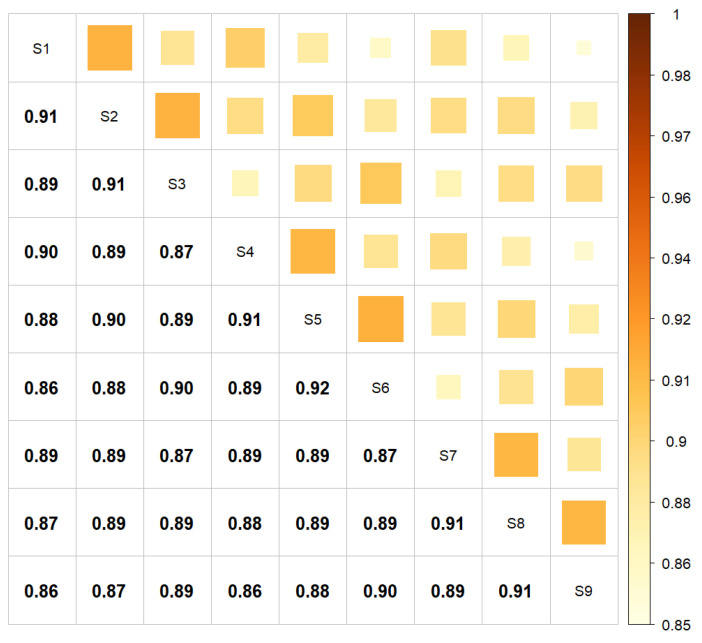
Degree of similitude between estimated haplotype phases in the nine scenarios of phasing.

**Haplotype Phasing.** The results of comparing haplotype phasing using nine combinations of core length and core tail parameters using Alphaphase software are presented in Figure 1.

The average degree of similitude was 0.89, and it was consistently above 0.86. Specifically, the predicted haplotype phase was identical across all nine scenarios for only 78.74% of the analyses but had concordance in more than seven scenarios for 92.5% of SNPs. These findings indicated that the output of the phasing algorithm was highly dependent on the specific set of parameters used for its implementation when medium-density SNP chips were used.

**Calculation of Gametic Matrices**. The diagonal values of the gametic matrices for the Entrepelado population ranged from 0.894 to 1.100, while for the Retinto population, they ranged from 0.901 to 1.179. Table 3 shows the distribution of the gametic relationships observed in the off-diagonal elements of the gametic matrices.

The calculated gametic matrices yielded results consistent with the familiar relationships of the individuals, as gametic relationships around 0.50 indicated that the individuals shared sire (or dam), while gametic relationships around 0.25 suggested that the sires (or dams) of the individuals were fullsibs.

**Variance Components.** The posterior mean and standard deviation estimate of the variance components are presented in Table 4.

**Figure 2:**
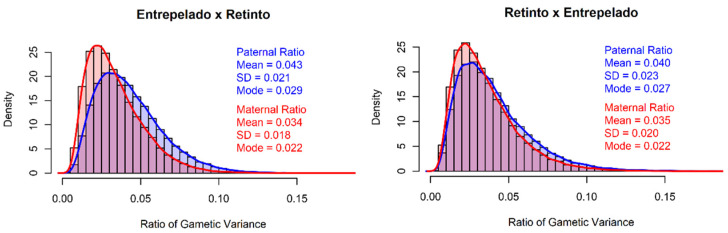
Posterior distributions of the ratio of gametic effect in the Entrepelado × Retinto and in the Retinto × Entrepelado crosses.

Furthermore, Figure 2 shows the posterior distributions of the ratios of gametic variances in the Entrepelado × Retinto (E × R) and Retinto × Entrepelado (R × E) crosses. The posterior mean estimates were similar, ranging between 0.034 for the Retinto maternal gametic effects in the ER cross and 0.043 for the Entrepelado paternal gametic effects in the RE cross.

These results indicate that there are no relevant differences in the amount of genetic variance contributed by the paternal and maternal origins in either of the two reciprocal crosses, based on the available information.

**Figure 3:**
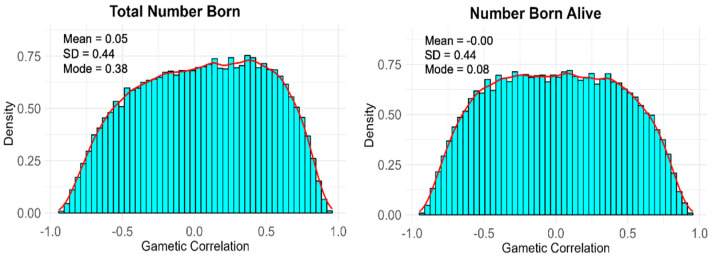
Posterior distributions of the gametic correlation between the paternal and maternal effects in the Entrepelado population.

**Figure 4:**
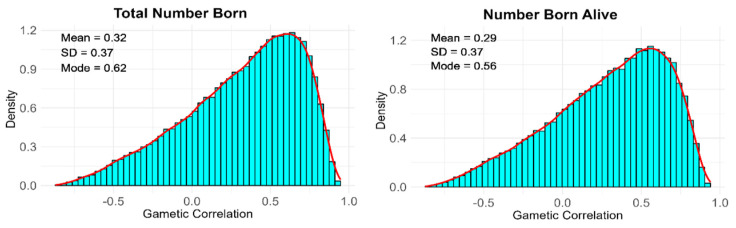
Posterior distributions of the gametic correlation between the paternal and maternal effects in the Retinto population.

**Gametic Correlations.** The posterior distribution of the gametic correlations for the TNB and NBA in the Entrepelado and Retinto populations are presented in Figure 3 and Figure 4, respectively.

The posterior distribution of the correlation between gametic effects in Retinto and Entrepelado showed notable differences in shape. Specifically, the posterior distributions of the gametic correlations in the Retinto population exhibited a higher degree of positive asymmetry compared to those in the Entrepelado population. In fact, the posterior probabilities of a positive gametic correlation in the Retinto population were 0.80 and 0.78 for the TNB and NBA, respectively. In contrast, the posterior probabilities of a positive gametic correlation in the Entrepelado population were 0.50 (TNB) and 0.54 (NBA).

Although caution is needed in interpreting the results due to the limited amount of phenotypic and genotypic information, the shape of the posterior distribution of gametic correlations suggests a potential role of genomic imprinting. This is because a gametic correlation substantially lower than one indicates that the same combination of alleles in a gamete may produce different effects on offspring depending on whether they are transmitted by paternal or maternal gametes, which is consistent with the theory of genomic imprinting. Genomic imprinting is an epigenetic phenomenon that causes genes to be expressed depending on whether they are inherited from the father or mother [7].

Several theories have been postulated to explain the evolutionary origin of genomic imprinting [15], and one of the most popular is the parental investment theory [16]. This theory argues that imprinting is the result of a conflict between the evolutionary success of paternally and maternally derived genes. In mammalian reproduction, the evolutionary success of paternally inherited genes is associated with the increase in fetal growth, while for maternally inherited genes, it is associated with the number of offspring. This theory is reinforced by the discovery of numerous imprinted genes known to regulate aspects of mammalian development [17], including growth, behavior, and placental function [18] and, furthermore, there is increasing evidence of imprinted genes in the pig genome [9,19,20].

From a practical perspective, a low or null gametic correlation between paternal and maternal gametes within the same population indicates that a selection program to improve the performance of the crossbreeding individuals needs to be specifically designed, especially in the Entrepelado population. This is because the selection of purebred animals to increase the performance in the Entrepelado × Retinto cross may not have any noticeable consequences in the performance in the Retinto × Entrepelado cross. Furthermore, this result also may explain the differences in performance among the reciprocal crosses observed by Noguera et al. [6], who proposed using the Retinto variety as a boar and the Entrepelado as a sow, providing better performance than the opposite cross.

## 4. Conclusions

The bivariate model proposed in this study provides estimates of the gametic effects of each founder population as either paternal or maternal, as well as their correlation. In the absence of parental imprinting, a perfect correlation of one would be expected. However, our results detect a significant deviation from this ideal scenario, indicating possible differences in the performance of crossbred individuals depending on the paternal or maternal origin of the gametes. These findings provide evidence of the presence of imprinting effects in Iberian pig populations, which could have implications for the design of future breeding programs.

## Figures and Tables

**Table 1:** The number of records (and sows between brackets), mean (±standard deviation) of the total number born and the number born alive, for Entrepelado × Retinto and Retinto × Entrepelado crosses.

	Entrepelado × Retinto	Retinto × Entrepelado
N ^1^ (NS) ^2^	1258 (203)	700 (125)
TNB ^3^	8.78 ± 2.24	8.85 ± 2.37
NBA ^4^	8.55 ± 2.23	8.62 ± 2.34

^1^ N: number of records. ^2^ NS: number of sows. ^3^ TNB: total number born. ^4^ NBA: number born alive.

**Table 2:** Parameters (core and tail length) in the nine scenarios of haplotype phasing.

Scenario	Core Length	Tail Length
S1	75	100
S2	75	150
S3	75	200
S4	100	100
S5	100	150
S6	100	200
S7	125	100
S8	125	150
S9	125	200

**Table 3:** Distribution of gametic relationships between the Entrepelado and Retinto gametic effects.

Gametic Relationship	Entrepelado	Retinto
**<0.05**	92,276 (86.03%)	94,144 (87.77%)
**0.05–0.10**	8130 (7.58%)	8900 (8.29%)
**0.10–0.20**	4670 (4.35%)	3130 (2.92%)
**0.20–0.30**	1076 (1.00%)	582 (0.54%)
**0.30–0.40**	480 (0.44%)	252 (0.23%)
**0.40–0.50**	396 (0.36%)	188 (0.18%)
**>0.50**	228 (0.21%)	60 (0.05%)

**Table 4:** Posterior mean (and standard deviation) of the variance components for the total number born, TNB, and the number born alive, NBA.

Variance Component	TNB	NBA
$\sigma_{s\left(ER\right)}^{2}$	0.144 (0.098)	0.142 (0.097)
$\sigma_{s\left(RE\right)}^{2}$	0.357 (0.187)	0.365 (0.191)
$\sigma_{p\left(E\right)}^{2}$	0.206 (0.103)	0.199 (0.100)
$\sigma_{m\left(E\right)}^{2}$	0.197 (0.114)	0.199 (0.115)
$\sigma_{p\left(R\right)}^{2}$	0.224 (0.132)	0.222 (0.128)
$\sigma_{m\left(R\right)}^{2}$	0.163 (0.087)	0.151 (0.080)
$\sigma_{e\left(ER\right)}^{2}$	4.296 (0.187)	4.251 (0.185)
$\sigma_{e\left(RE\right)}^{2}$	4.795 (0.288)	4.607 (0.278)

## Data Availability

The dataset used in this study will be available upon reasonable request to the corresponding author (lvarona@unizar.es).

## References

[1] Serra X., Gil F., Pérez-Enciso M., Oliver M.A., Vázquez J.M., Gispert M., Díaz I., Moreno F., Latorre R., Noguera J.L. (1998). A comparison of carcass, meat quality and histochemical characteristics of Iberian (Guadyerbas line) and Landrace pigs. Livest. Prod. Sci..

[2] Serrano M.P., Valencia D.G., Nieto M., Lázaro R., Mateos G.G. (2008). Influence of sex and terminal sire line on performance and carcass and meat quality of Iberian pigs reared under intensive production systems. Meat Sci..

[3] Silio L., Rodriguez M.C., Rodrigañez J., Toro M.A., Buxade C., Daza A. (2001). La selección de cerdos ibéricos. Porcino Ibérico: Aspectos Claves.

[4] Fernández A., Rodrigáñez J., Zuzúarregui J., Rodríguez M.C., Silió L. (2008). Genetic parameters for litter size and weight at different parities in Iberian pigs. Span. J. Agric. Res..

[5] García-Casco J.M., Fernández A., Rodríguez M.C., Silió L. (2012). Heterosis for litter size and growth in crosses of four strains of Iberian pig. Livest. Sci..

[6] Noguera J.L., Ibáñez-Escriche N., Casellas J., Rosas J.P., Varona L. (2019). Genetic parameters and direct, maternal and heterosis effects on litter size in a diallel cross among three commercial varieties of Iberian pig. Animal.

[7] Reik W., Walter J. (2001). Genomic imprinting: Parental influence on the genome. Nat. Rev. Genet..

[8] Hanna C.W. (2020). Placental imprinting: Emerging mechanisms and functions. PLoS Genet..

[9] Coster A., Madsen O., Heuven H.C.M., Dibbits B., Groenen M.A.M., van Arendonk J.A.M., Bovenhuis H. (2012). The imprinted gene DIO3 is a candidate gene for litter size in pigs. PLoS ONE.

[10] Nishio M., Satoh M. (2015). Genomic best linear unbiased prediction method including imprinting effects for genomic evaluation. Genet. Sel. Evol..

[11] Hickey J.M., Kinghorn B.P., Tier B., Wilson J.F., Dunstan N., Van Der Werf J.H.J. (2011). A combined long-range phasing and long haplotype imputation method to impute phase for SNP genotypes. Genet. Sel. Evol..

[12] Srihi H., Ibáñez-Escriche N., Casellas J., Noguera J.L., Hernández P., Martín de Hijas M., Vázquez-Gómez M., Negro S., Rosas J.P., Varona L. Bayesian analysis of paternal and maternal gametic effects in a reciprocal cross between two Iberian varieties. Proceedings of the 12th World Congress on Genetics Applied to Livestock Production.

[13] Gelfand A.E., Smith A.F.M. (1990). Sampling-Based Approaches to Calculating Marginal Densities. J. Am. Stat. Assoc..

[14] Misztal I., Tsuruta S., Lourenco D., Aguilar I., Legarra A., Vitezica Z. (2018). Manual for BLUPF90 Family of Programs.

[15] Patten M.M., Ross L., Curley J.P., Queller D.C., Bonduriansky R., Wolf J.B. (2014). The evolution of genomic imprinting: Theories, predictions and empirical tests. Heredity.

[16] Moore T., Haig D. (1991). Genomic imprinting in mammalian development: A parental tug-of-war. Trends Genet..

[17] Thamban T., Agarwaal V., Khosla S. (2020). Role of genomic imprinting in mammalian development. J. Biosci..

[18] Fowden A.L., Coan P.M., Angiolini E., Burton G.J., Constancia M. (2011). Imprinted genes and the epigenetic regulation of placental phenotype. Prog. Biophys. Mol. Biol..

[19] Wu Y.Q., Zhao H., Li Y.J., Khederzadeh S., Wei H.J., Zhou Z.Y., Zhang Y.P. (2020). Genome-wide identification of imprinted genes in pigs and their different imprinting status compared with other mammals. Zool. Res..

[20] Zhang F.W., Han Z.B., Deng C.Y., He H.J., Wu Q. (2012). Conservation of genomic imprinting at the NDN, MAGEL2 and MEST loci in pigs. Genes Genet. Syst..

